# Synthesis of trifunctional cyclo-β-tripeptide templates

**DOI:** 10.3762/bjoc.8.180

**Published:** 2012-09-19

**Authors:** Frank Stein, Tahir Mehmood, Tilman Plass, Javid H Zaidi, Ulf Diederichsen

**Affiliations:** 1 Institute for Organic and Biomolecular Chemistry, University of Göttingen, Tammannstrasse 2, D-37077 Göttingen, Germany; 2 Department of Chemistry, Quaid-i-Azam University, Islamabad, 45320, Pakistan

**Keywords:** β-amino acids, cyclic β-tripeptide scaffold, orthogonal protection groups, peptide synthesis, template-assembled synthetic proteins (TASP)

## Abstract

The concept of template-assembled synthetic proteins (TASP) describes a central scaffold that predefines the three dimensional structure for diverse molecules linked to this platform. Cyclic β-tripeptides are interesting candidates for use as templates due to their conformationally defined structure, stability to enzymatic degradation, and ability to form intermolecular stacked tubular structures. To validate the applicability of cyclic β-tripeptides within the TASP concept, an efficient synthesis of the cyclopeptide with orthogonal functionalization of the side chains is desired. A solid-phase-supported route with on-resin cyclization is described, employing the aryl hydrazide linker cleavable by oxidation. An orthogonal protection-group strategy allows functionalization of the central cyclic β-tripeptide with up to three different peptide fragments or fluorescent labels.

## Introduction

**Figure 1:**
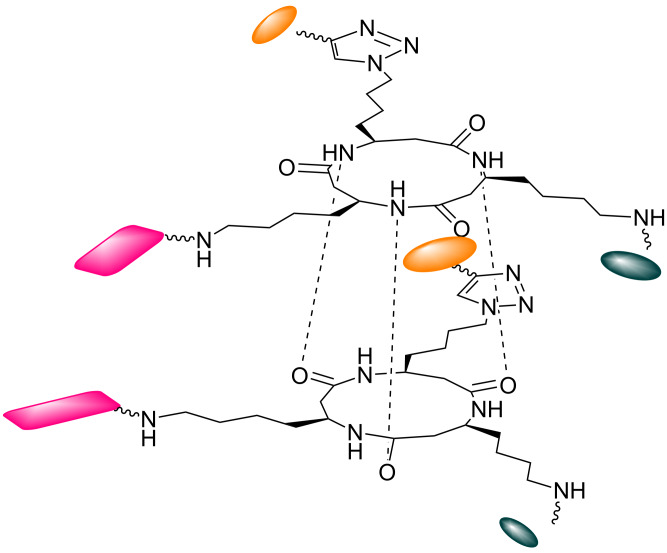
Trifunctional cyclic β-tripeptide forming an intermolecular stack of rings by backbone hydrogen bonding.

Cyclic β-tripeptides form structurally well-defined secondary structures with the potential for alignment of the rings to form intermolecular aggregates [1]. Due to the unidirectional alignment of the carbonyl groups and the flattened-ring conformation, cyclic β-tripeptides form assemblies of stacked rings through hydrogen bonding [2]. Furthermore, they are exceedingly stable against proteolytic cleavage and enzymatic degradation [3–4]. These properties make cyclic β-tripeptides interesting candidates for the concept of template-assembled synthetic proteins (TASP) [5–6]. The TASP concept describes a central scaffolding molecule directing all further attached molecules into a spatially predefined structure. Cyclic β-tripeptides are suitable for use as a central scaffold that carries different kinds of molecules and functionalities to direct them into a trigonal planar assembly. This idea was already used for the synthesis of a *C*_3_-symmetric ligand for the immune response receptor CD40 [7]. However, the synthetic route to these kinds of molecules is demanding due to solution-phase synthesis of the β-tripeptides and their final cyclization with low efficiency [8–9]. Moreover, only homofunctionalized cyclic β-tripeptides have been described so far [9–10]. To further investigate and exploit the potential of this class of circular peptides, a synthetic route should fulfil the requirements of (i) fast synthetic access and (ii) the possibility to attach different molecules on each side chain of the central scaffold. Here we report a new effective synthesis for cyclic β-tripeptides on a solid support, employing the oxidation-labile aryl hydrazide linker [11]. We also describe an orthogonal protection-group strategy to synthesize a trifunctional cyclic β-tripeptide that has the potential of forming intermolecular hydrogen-bonded stacks (Figure 1).

## Results and Discussion

### General strategy

To equip the cyclic β-tripeptide with three different functional units, each amino acid residue needs to be specifically addressed. Furthermore, all three amino acids should have side chains long enough to avoid steric hindrance between the moieties and the core. Thus, homo-β-lysine was chosen as the underlying amino acid to build up the scaffold allowing side-chain functionalization by amide bond formation. To protect the lysine side chain for selective and orthogonal amide-bond formation following the solid-phase peptide synthesis (SPPS), the protection groups fluorenylmethoxycarbonyl (Fmoc) and carbobenzyloxy (Cbz) were applied. Alteration of the amine in the third β-homolysine side chain to an azide [12–14] employing the Wong azidation [15] enables Huisgen [3 + 2]-cycloaddition [16] as an orthogonal coupling method. In order to build up the peptide sequence in the presence of Fmoc, Cbz and the azide, an acid-labile protecting group was required for temporary protection of the primary α-amino group. Therefore, *tert*-butyloxycarbonyl (Boc) protection was used to mask all α-amino groups during solid-phase synthesis of the tripeptide.

#### Synthesis of the cyclo-β-peptide scaffold

**Figure 2:**
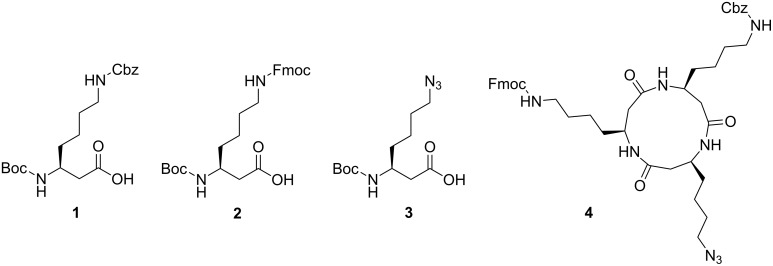
β-Amino acids **1**–**3** with orthogonal side-chain protection obtained by Arndt–Eistert homologation followed by Wolff rearrangement; cyclo-β-tripeptide template **4** as obtained by coupling of amino acids **1**–**3** followed by cyclization.

The Boc protected β-amino acid building blocks for SPPS of the cyclic β-tripeptide were prepared from the respective β-amino acids by Arndt–Eistert homologation [17–19]. The β-amino acids were transformed into the respective diazoketones with isobutyl chloroformate, triethylamine and diazomethane. The ketones were further converted into the β-amino acids by Wolff rearrangement using silver benzoate and water as a nucleophile [19–23] yielding the homo-β-lysine derivatives **1** and **2** alongside the azide β-amino acid **3** (Figure 2).

In previous studies, synthesis of the cyclic β-tripeptide scaffold was provided by cyclization of the linear β-tripeptide obtained by solution-phase chemistry [8–9]. Purification by chromatography is required after each coupling step, and the final cyclization reaction often results in poor yields. Herein, an alternative approach is described based on SPPS followed by on-resin cyclization. Recently, Waldmann et al. discovered an on-resin head-to-tail cyclization based on a Boc protocol using the oxidation-labile aryl hydrazide linker [24] and cleavage from the resin under simultaneous cyclization [11]. This method was adapted for the synthesis of the cyclic β-tripeptide **4** (Figure 2).

**Scheme 1:**
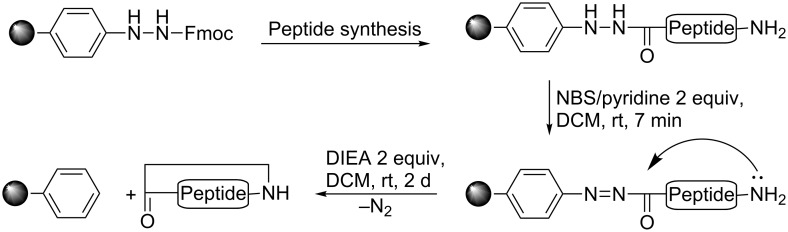
Synthesis of cyclic peptides employing the oxidation-labile aryl hydrazide linker [11,24].

The synthesis of the cyclic β-tripeptide was performed according to Scheme 1 by using the commercially available 4-Fmoc-hydrazinobenzoyl AM NovaGel resin from Merck Biosciences. After coupling of the three β-amino acids by using the standard Boc-protocol, the hydrazide linker was oxidized to generate nitrogen as a good leaving group. Nucleophilic attack of the N-terminal amino group at the activated carbonyl group provided cleavage of the cyclized β-tripeptide from the resin. Purification did not require any chromatography since organization by intermolecular hydrogen bonding resulted in decreased solubility and precipitation of the β-tripeptide template **4** from methanol.

#### Functionalization of the β-tripeptide template

**Figure 3:**
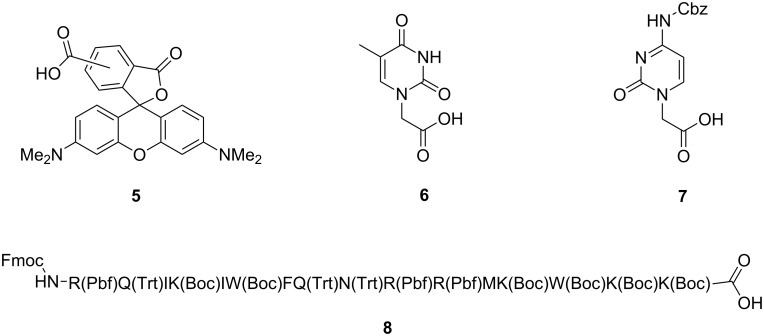
Functional units provided as carboxylic acids for the attachment to the cyclo-β-peptide: 5(6)-tetramethylcarboxyrhodamine **5**, thymine **6**, Cbz protected cytosine with a carboxylic acid linker **7** [25], and fully protected peptide sequence of penetratin **8** [26].

The cyclic β-tripeptide template **4** has the potential for orthogonal functionalization at the side chains with up to three different moieties, by successive amide-bond formation and by employing the Huisgen [3 + 2]-cycloaddition. Aggregation by stacking of the functionalized peptide rings will further provide a higher density of organized recognition motifs and labels. As a proof-of-concept, the successive coupling of a fluorophore 5(6)-tetramethylcarboxyrhodamine **5** (TAMRA-COOH) and a cell-penetrating peptide to the template **4** is reported, as well as the functionalization with the nucleobase recognition units thymine-1-yl acetic acid (**6**) and (*N*^4^-benzyloxycarbonyl)cytosine-1-yl acetic acid (**7**) (Figure 3). In all cases, the template contains a third option for functionalization by covalent attachment of molecules through [3+2]-cycloaddition under mild conditions.

The template approach allows for the preparation of constructs that, e.g., combine biological activity, fluorescence labelling and cell-penetrating or cell-directing units in one molecule to be used for in vivo studies. The defined spatial organization of recognition units such as nucleobases on the template provides a specific hydrogen-bonding network orthogonal to the peptide ring aggregation, which will be of value for aggregate formation and molecular architectures defined by a three-dimensional network of hydrogen bonds.

**Scheme 2:**
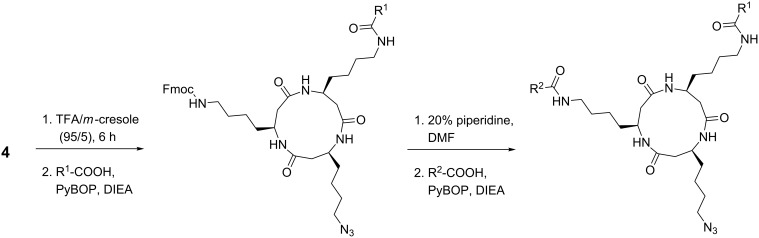
Functionalization of the cyclic β-tripeptide **4**.

**Figure 4:**
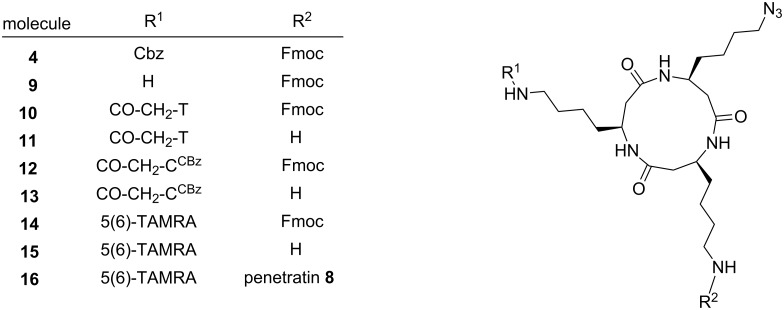
Cyclic β-tripeptides varying in two side-chain functionalities and containing an additional azide moiety, e.g., for ligation with click chemistry.

Selective release of the first amino group was achieved by removal of the Cbz protecting group using TFA and *m*-cresol (95:5 v/v). The first functional unit of a recognition moiety was attached to the template as a carboxylic acid by amide-bond formation using PyBOP and DIEA, or PyBrOP and DIEA for coupling (Scheme 2). Exemplarily, TAMRA-COOH (**5**) and the nucleobase moieties thymine-1-yl acetic acid (**6**) and (*N*^4^-benzyloxycarbonyl)cytosine-1-yl acetic acid (**7**) were attached to the cyclo-β-peptide yielding monofunctionalized templates **14**, **10** and **12** (Figure 4).

The second amino group was Fmoc-deprotected with 20% piperidine in DMF. Coupling of the fully protected cell-penetrating peptide penetratin **8** [26] with the homolysine side chain of the cyclic β-tripeptide **15** was accomplished by HOBt and HBTU activation in solution using a 10-fold access of penetratin. The β-peptide template **16** functionalized at two side chains was obtained, purified by HPLC, and characterized by mass spectrometry.

## Conclusion

Cyclic β-tripeptides provide an interesting platform for the concurrent arrangement of side-chain bound functionalities in combination with the ability of intermolecular tubular aggregation of the rings. An easy and fast approach towards cyclic β-tripeptides was presented. The tripeptide scaffold was assembled completely on a solid support, thereby saving purification steps following amino acid coupling. Moreover, cleaving of the peptide from the resin and cyclization was performed in a single step employing the oxidation-labile aryl hydrazide linker [11]. Together with a convenient and efficient purification procedure, this method is beneficial with respect to yield and speed of the synthesis. Further, an orthogonal protection strategy was introduced to equip the β-peptide template with three different functional molecules allowing testing of the cyclic β-tripeptides as a scaffold for the defined orientation of functional units and the combination of up to three modules in one molecule. The advantages of using the β-peptide template within the TASP concept are the spatially defined and rigid structure, with a high stability against enzymatic degradation, and their ability to form intermolecular staples by backbone hydrogen bonding. This might be of special advantage to target or imitate multivalent and/or cooperative processes.

As an initial effort, we equipped the cyclic β-tripeptide with a fluorophore and a protected cell-penetrating peptide, which can be further functionalized with any biologically active molecule bearing an alkyne. Potentially, this generates a fluorescent and cell-permeable drug applicable for in vivo experiments and other biochemical assays. In addition, different nucleobases were linked to the central core in order to generate a template that can form a three dimensional network of hydrogen bonds.

## Experimental

### General remarks

All technical solvents were distilled prior to use. The solvents of analytical and HPLC grade were used as supplied. The solvents used for the synthesis were obtained in quality puriss. abs. from Acros Organics, Sigma Aldrich, Merck, or VWR. All chemicals were of the highest grade available and used as supplied. All moisture- and oxygen-sensitive reactions were carried out under an inert gas atmosphere (nitrogen or argon). Analytical TLC was performed on Merck TLC aluminium sheets silica gel 60 F_254_. Detection was performed under UV light (254 nm) or by dipping into a solution of ninhydrin (3% in ethanol) followed by heating with a heat gun. Eluents and the appropriate *R*_f_ values are indicated. The columns for flash chromatography were packed with silica gel 60 from Macherey–Nagel with a grit size of 0.063 to 0.2 mm and were run under a pressure of 1 to 1.5 bar. The substance was applied as a concentrated solution or adsorbed on silica gel. Eluents are indicated. Reverse-phase HPLC was performed on an Äkta Basic 900 from Pharmacia Biotech. UV detection was performed at 215, 254 and 280 nm wavelength. The solvents used for HPLC were of HPLC grade and degassed while being stirred in vacuo. Demineralized Water for HPLC use was preprocessed by the water treatment plant “Simplicity” from Millipore. All HPLC runs were performed by using linear gradients between A (0.1% aq TFA), B (0.1% TFA in methanol) or C (0.1% TFA in MeCN) and water (0.1% TFA) within 30 minutes. Flow rates were taken as 1 mL/min for the analytical columns and 10 mL/min for preparative columns. All crude samples were dissolved in methanol or acetonitrile and filtered prior to use. Electrospray ionization (ESI) mass spectra were obtained on a Finnigan LCQ instrument. High-resolution mass spectra (HRMS) were obtained on a Bruker Apex IV FT-ICR-MS instrument. ^1^H NMR and ^13^C NMR spectra of samples dissolved in DMSO-*d*_6_, CDCl_3_ or acetone-*d*_6_ were recorded on a Varian Unity 300 (300 MHz) spectrometer. Residual solvent proton signals were used as an internal standard.

#### Synthesis of compounds

**(*S*)-*tert*-Butyl(7-azido-1-diazo-2-oxoheptan-3-yl)carbamate.** (*S*)-6-Azido-2-(*tert*-butoxycarbonylamino)hexanoic acid (7.38 g, 27.1 mmol, 1.00 equiv) was dissolved in dry THF (108 mL) under an argon atmosphere. The solution was cooled at −15 °C and dry triethylamine (4.19 mL, 29.8 mmol, 1.10 equiv) and isobutyl chloroformate (3.91 mL, 29.8 mmol, 1.10 equiv) were slowly added and the whole mixture was stirred for 45 min. The reaction mixture was warmed to 0 °C, and under exclusion of light an approximately 0.35 M solution of diazomethane (181 mL, 54.2 mmol, 2.00 equiv) in Et_2_O was added. After 15 min, the reaction mixture was stirred for an additional 6 h at room temperature. Excess diazomethane was decomposed by dropwise addition of AcOH. The solvent was removed under reduced pressure, and the residue was dissolved in Et_2_O and washed with saturated solutions of NaHCO_3_ (3 × 50 mL), NH_4_Cl (2 × 50 mL) and NaCl (1 × 50 mL). The combined organic layers were dried over MgSO_4_, filtered and concentrated under reduced pressure to furnish a yellow oil. The title compound was used for the next reaction without further purification. DC (EtOAc/*n*-pentane 2:3 + 1% AcOH): *R*_f_ = 0.73.

**(*S*)-7-Azido-3-(*tert*-butoxycarbonylamino)heptanoic acid (3).** A solution of silver trifluoroacetate (219 mg, 990 µmol, 0.110 equiv) in triethylamine (3.79 mL, 27.0 mmol, 3.00 equiv) was slowly added to a solution of (*S*)-*tert*-butyl 7-azido-1-diazo-2-oxoheptan-3-yl carbamate (2.67 g, 9.00 mmol, 1.00 equiv) in THF/H_2_O 9:1 (45.0 mL) under exclusion of light at −15 °C. The reaction solution was warmed to room temperature after 30 min and stirred for a further 12 h until completion. The solvent was removed under reduced pressure. The residue was dissolved in Et_2_O and washed with a saturated solution of NaHCO_3_ (3 × 50 mL). The combined aqueous layers were acidified with 2 M hydrochloric acid to pH 2–3 and were extracted with EtOAc (5 × 50 mL). The combined organic layers were dried over MgSO4, filtered and concentrated under reduced pressure. The title compound was obtained after purification by flash chromatography (silica gel, 400 g, DCM/MeOH 9:1 + 3% AcOH) to furnish a yellow oil (0.714 g, 2.49 mmol, 28% yield). DC (DCM/MeOH 9:1, 3% AcOH): *R*_f_ = 0.51; ^1^H NMR (300 MHz, acetone-*d*_6_) δ 1.38 (s, 9H, Boc-CH_3_), 1.42–1.70 (m, 6H, γ-H2, δ-H2, ε-H2), 2.41–2.56 (m, 2H, -H2), 3.30–3.38 (t, ^3^*J*_H,H_ = 6.6 Hz, 2H, ζ-H2), 3.93 (m, 1H, β-H), 5.89 (d, ^3^*J*_H,H_ = 7.2 Hz, 1H, NHBoc); ESIMS *m*/*z*: 309.2 [M + Na]^+^, 285.2 [M − H]^−^; HRMS: C_12_H_22_N_4_O_4_: [M + Na]^+^ calcd: 309.1533; found: 309.1534, [M − H]^−^ calcd: 285.1568; found: 285.1565.

**Fmoc-R(Pbf)Q(Trt)IK(Boc)IW(Boc)FQ(Trt)N(Trt)R(Pbf)R(Pbf)MK(Boc)W(Boc) K(Boc)K(Boc)-OH (8).** The fully protected penetratin **8** was synthesized on H-Lys(Boc)-2-ClTrt resin (0.61 mmol/g, 163.9 mg, 0.100 mmol, 1.00 equiv) using a Liberty peptide synthesizer (CEM*,* Kamp-Lintfort, Germany) equipped with a Discover microwave reaction cavity (CEM). Standard reagents, protocols and procedures were used for deprotection (20% piperidine in NMP, 210 s, 50 °C, 20 W) and coupling (HBTU/HOBt/DIEA/NMP, 300 s, 50 °C, 20 W). Double couplings were performed for arginine. No final Fmoc deprotection step was used. After the automated peptide synthesis, the peptide was cleaved from the resin using a 30% solution of HFIP in DCM (5.00 mL) for 45 min. Afterwards, the cleavage cocktail was filtered, the resin was washed (3 × 3 mL 30% HFIP in DCM) and all phases combined. The excess solvent was removed under a nitrogen stream and the crude product was precipitated by adding MTBE (10 mL). The title compound was obtained after RP-HPLC purification to yield a white solid (117 mg, 25.6 µmol, 26% yield); RP-HPLC: *t*_R_ = 34.7 min (98→100% C in 40 min); ESIMS *m*/*z*: 4552.28 [M]^−^; HRMS: C_245_H_316_N_34_O_43_S_4_ [M]^+^ calcd: 4550.25; found: 4550.29.

**cyclo(β^3^-HLys(Fmoc)-β^3^-HLys(N_3_)-β^3^-HLys(Cbz)) (4).** The peptide sequence H-β^3^-HLys(Fmoc)-β^3^-HLys(N_3_)-β^3^-HLys(Cbz)-OH was synthesized on 4-Fmoc-hydrazinobenzoyl AM NovaGel resin (0.700 mmol/g, 1.00 g, 7.00 mmol, 1.00 equiv). After N-terminal Fmoc deprotection (20% piperidine in DMF, 3.50 mL, 20 min), standard Boc protocols were used for coupling (β-amino acid 5.00 equiv, HBTU 4.50 equiv, HOBt 5.00 equiv, DIEA 10.0 equiv, 2.80 mL DMF, 18 h) and Boc cleavage (3.00 mL TFA/*m*-cresole–95/5 v/v, 2 × 2 min) of β-amino acids Boc-β^3^-HLys(Fmoc)-OH, Boc-β^3^-HLys(Cbz)-OH and Boc-β^3^-HLys(N_3_)-OH. After N-terminal deprotection of the last amino acid, the resin was washed with DCM (3 × 3 mL) and the hydrazinobenzoyl linker was oxidized to the acyldiazene by stirring the resin in a solution of *N*-bromosuccinimide (0.249 g, 1.40 mmol, 2.00 equiv) and pyridine (113 μL, 1.40 mmol, 2.00 equiv) in DCM (3.50 mL) for 7 min. The resin was washed with DCM (3 × 3 mL). Then, a solution of DIEA (244 μL, 1.40 mmol, 2.00 equiv) in DCM (3.50 mL) was added to the resin and the reaction mixture was stirred for 48 h. The resin was filtered and washed with DCM (5 × 3.00 mL).The washing fractions were combined and the organic solvent was removed under reduced pressure. The title compound was obtained after recrystallization (MeOH, 3.00 mL) to furnish a yellow powder (114 mg, 141 μmol, 29% yield). DC (DCM/MeOH 9:1): *R*_f_ = 0.42; ESIMS *m*/*z*: 831.4 [M + Na]^+^; HRMS: C_44_H_56_N_8_O_7_ [M + Na]^+^ calcd: 831.4164; found: 831.4171.

**cyclo(β^3^-HLys(Fmoc)-β^3^-HLys(N_3_)-β^3^-HLys) (10).** The completely protected cyclic β-tripeptide **4** (79.4 mg, 98.0 μmol) was dissolved in a mixture TFA and *m*-cresol (95:5 v/v 5 mL) and stirred for 6 h. The crude peptide was precipitated with ice-cold MTBE (3 × 13 mL) and the title compound was obtained by RP-HPLC purification to give a yellow solid (43.2 mg, 64.0 μmol, 65% yield). RP-HPLC: *t*_R_ = 23.20 min (40→100% B in 30 min); ESIMS *m*/*z*: 675.4 [M]^+^; HRMS: C_26_H_50_N_8_O_5_ [M]^+^ calcd: 675.3977; found: 675.3974.

**cyclo(β^3^-HLys(Fmoc)-β^3^-HLys(N_3_)-β^3^-HLys(TAMRA)) (14).** The cyclic β-peptide **9** (43.2 mg, 64.0 μmol, 1.00 equiv), PyBOP (48.3 mg, 93.0 μmol, 1.45 equiv) and 5(6)-TAMRA (40.0 mg, 93.0 μmol, 1.45 equiv) were dissolved in DMF (500 μL) and DIEA (32.0 μL, 186 μmol, 2.90 equiv) was added. The reaction mixture was for 6 h at room temperature. The crude peptide was precipitated with ice-cold MTBE (3 × 14 mL) and the title compound was obtained by RP-HPLC purification to give a pink solid (39.4 mg, 36.0 μmol, 57% yield). RP-HPLC: *t*_R_ = 22.95→23.43 min (40→90% B in 30 min); ESIMS *m*/*z*: 1087.4 [M]^+^, 1109.4 [M + Na]^+^; HRMS: C_61_H_70_N_10_O_9_ [M]^+^ calcd: 1087.5400; found: 1087.5419.

**cyclo(β^3^-HLys-β^3^-HLys(N_3_)-β^3^-HLys(TAMRA)) (15).** The cyclo-β-tripeptide **14** (39.4 mg, 36.0 μmol, 1.00 equiv) was dissolved in a solution of 20% piperidine in DMF (500 μL) and stirred for 3 min at 50 °C (25 W) by using a manual discover SPS ultrasound peptide synthesizer. Afterwards, the crude peptide was precipitated with ice-cold MTBE (1 × 15 mL) and the title compound was obtained after RP-HPLC purification (23.2 mg, 27.0 μmol, 74% yield). RP-HPLC: *t*_R =_ 14.50→15.34 min (40→95% B in 30 min); ESIMS *m*/*z*: 433.2 [M + 2H]^2+^, 865.4 [M]^2+^; HRMS: C_46_H_60_N_10_O_7_ [M]^+^ calcd: 865.4719; found: 865.4718.

**cyclo(β^3^-HLys(penetratin 8)-β^3^-HLys(N_3_)-β^3^-HLys(TAMRA)) (16).** The fully protected peptide penetratin **8** (9.00 mg, 1.98 μmol, 1.43 equiv) was dissolved in DMF (30.0 μL) and DIEA (2.42 μL, 14.0 μmol, 10.0 equiv) and stock solutions of HOBt and HBTU in DMF (4.16 μL, 2.08 μmol, 1.50 equiv) were added. After 5 min of preactivation, the solution was added to the cyclic β-tripeptide **15** (1.20 mg, 1.39 μmol, 1.00 equiv) and stirred under exclusion of light for 48 h. The crude product was precipitated with ice-cold MTBE (1 × 14 mL) and the title compound was obtained by RP-HPLC purification to give a pink solid (1.38 mg, 0.256 μmol, 18% yield). For spectrometry, a small portion was deprotected by TFA for 2 h, precipitated with ice-cold MTBE and analyzed by mass spectrometry. RP-HPLC: *t*_R_ = 31.8 min (95→100% B in 40 min). Deprotected compound: ESIMS *m*/*z*: 3314.83 [M + H]^+^; HRMS: C_165_H_236_N_44_O_28_S [M]^+^ calcd: 3313.81; found: 3313.83.

**cyclo(β^3^-HLys(Fmoc)-β^3^-HLys(N_3_)-β^3^-HLys-(thymin-1-yl acetate)) (10).** Cyclo(β^3^-HLys(Fmoc)-β^3^-HLys(N_3_)-β^3^-HLys) (**9**, 6.24 mg, 9.25 µmol, 1.00 equiv) was dissolved in dry NMP (700 µL). Thymin-1-ylacetic acid **6** (8.51 mg, 46.3 µmol, 5.01 equiv), PyBrOP (21.6 mg, 46.3 µmol, 5.01 equiv) and DIEA (16.1 µL, 92.5 µmol, 10.0 equiv) were added. After stirring of the reaction mixture for 72 h at room temperature, the solvent was evaporated under reduced pressure and the crude title compound (6.61 mg, 7.86 µmol, 85%) was recrystallized from a mixture of MeOH/TFA (4:1, 1.00 mL) to yield a brownish solid. The crude product was directly used in the next step without further purification. ESIMS *m*/*z*: 863.4 [M + Na]^+^_;_ HRMS: C_43_H_56_N_10_O_8_ [M + Na]^+^ calcd: 863.4175; found: 863.4174, [M − H]*^−^* calcd: 839.4210; found: 839.4214.

**cyclo(β^3^-HLys(NH_2_)-β^3^-HLys(N_3_)-β^3^-HLys(thymin-1-yl acetate)) (11).** Cyclo(β^3^-HLys(Fmoc)-β^3^-HLys(N_3_)-β^3^-HLys-(thymin-1-yl acetate)) (**10**, 7.61 mg, 9.05 µmol, 1.00 equiv) was dissolved in a solution of 20% piperidine in DMF (1000 µL). The reaction mixture was sonicated for 35 min in an ultrasonic cleaning bath. Then, ice-cold *tert*-butylmethylether (MTBE) (14 mL) was added. The observed precipitate was separated by centrifugation (20 min, −5 °C, 9000 rpm) and the residue was dried in high vacuum. The title compound was obtained by RP-HPLC purification (5.04 mg, 8.15 µmol, 90%) to obtain a brownish solid. RP-HPLC: *t*_R_ = 10.5 min (30→100% B in 30 min); ESIMS *m*/*z*: 641.4 [M + Na]^+^; HRMS C_28_H_46_N_10_O_6_: [M + H]^+^ calcd: 619.3635; found: 619.3637, [M + Na]^+^ calcd: 641.1658; found: 641.1665.

**cyclo(β^3^-HLys(Fmoc)-β^3^-HLys(N_3_)-β^3^-HLys((*N*^4^-benzyloxycarbonyl) cytosine-1-yl acetate)) (12).** Cyclo(β^3^-HLys(Fmoc)-β^3^-HLys(N_3_)-β^3^-HLys) (**9**, 12.5 mg, 18.5 µmol, 1.00 equiv) was dissolved in dry NMP (1000 µL). Then, (*N*^4^-benzyloxycarbonyl) cytosine-1-yl acetic acid **7** (28.1 mg, 92.5 µmol, 5.00 equiv), bromo-tris-pyrrolidino phosphonium hexafluorophosphate (PyBrOP) (43.2 mg, 92.6 µmol, 5.01 equiv) and DIEA (32.2 µL, 185 µmol, 10.0 equiv) were added. After stirring of the reaction mixture for 72 h, the solvent was removed under reduced pressure and the crude title compound (15.5 mg, 16.1 µmol, 87%) was recrystallized from a mixture of MeOH/TFA (9:1, 5.00 mL) to yield a white solid. The crude product was directly used in the next step without further purification. ESIMS *m*/*z*: 982.4 [M + Na]^+^; HRMS C_50_H_61_N_11_O_9_: [M + H]^+^ calcd: 960.4726; found: 960.4708, [M + Na]^+^ calcd: 982.4546; found: 982.4527.

**Synthesis of cyclo(β^3^-HLysNH_2_-β^3^-HLys(N_3_)-β^3^-HLys((*N*^4^-benzyloxycarbonyl) cytosine-1-yl acetate) (13).** Cyclo(β^3^-HLys(Fmoc)-β^3^-HLys(N_3_)-β^3^-HLys-((*N*^4^-benzyloxycarbonyl) cytosine-1-yl acetic acid) (**12**, 15.0 mg, 15.6 µmol, 1.00 equiv) was dissolved in a solution of 20% piperidine in DMF (1000 µL). The reaction mixture was sonicated for 35 min in an ultrasonic cleaning bath. Then, ice-cold *tert*-butylmethylether (MTBE) was added. The observed precipitate was separated by centrifugation (15 min, 4 °C, 4500 rpm) and the residue was dried in high vacuum. The title compound was obtained by RP-HPLC purification (10.3 mg, 14.0 µmol, 90%) as a white solid. RP-HPLC: *t*_R_ = 8.8 min (10→40% B in 30 min); ESIMS *m*/*z*: 760.4 [M + Na]^+^; HRMS C_35_H_51_N_11_O_7_: [M − H]^−^ calcd: 736.3900; found: 736.3888, [M + Na]^+^ calcd: 760.3865; found: 760.3866.
